# Enhancing the safety and efficacy of cell therapy with programmed sense‐and‐respond function

**DOI:** 10.1002/ctm2.70328

**Published:** 2025-04-28

**Authors:** Andrew J. Walters, Xiaoyu Yang, Scott D. Olson, Caleb J. Bashor

**Affiliations:** ^1^ Department of Bioengineering Rice University Houston Texas USA; ^2^ Department of Pediatric Surgery McGovern Medical School University of Texas Health Science Center at Houston Houston Texas USA; ^3^ Department of Biosciences Rice University Houston Texas USA

## 1 MAIN TEXT

Over the past decade, cell‐based therapies have emerged as a transformative pharmaceutical modality, offering unprecedented potential for treating previously incurable diseases.^1^ Whilst most cell‐based therapies rely on intrinsic cell properties to achieve their therapeutic effects, genetic modification has gained traction as a strategy to enhance treatment safety and efficacy.^2^, ^3^ Amongst the most impactful therapeutic advancements are genetic engineering of adoptive T cell therapeutics, particularly for liquid tumour malignancies.^4^ Key breakthroughs include the development of chimeric antigen receptors (CARs) that reprogram T cell cytotoxicity towards tumour cells,^5^ protein‐based safety switches that trigger apoptosis upon administration of a small‐molecule drug^6^ and, most recently, synthetic multi‐gene circuits that enable T cells to detect tumour antigens or soluble factors and conditionally deliver anti‐tumour or immunomodulatory payloads in response.^7^ The continued evolution of this dynamic cell technology for broader clinical applications hinges on ongoing engineering innovations that enhance circuit precision and expand target detection capabilities. Towards this goal, we recently reported a circuit engineering toolkit that uses phosphorylation to drive circuit function, opening the door to engineering therapeutic sense‐and‐respond functionality that operates with the speed and precision of native cellular signalling pathways.^8^

**FIGURE 1:**
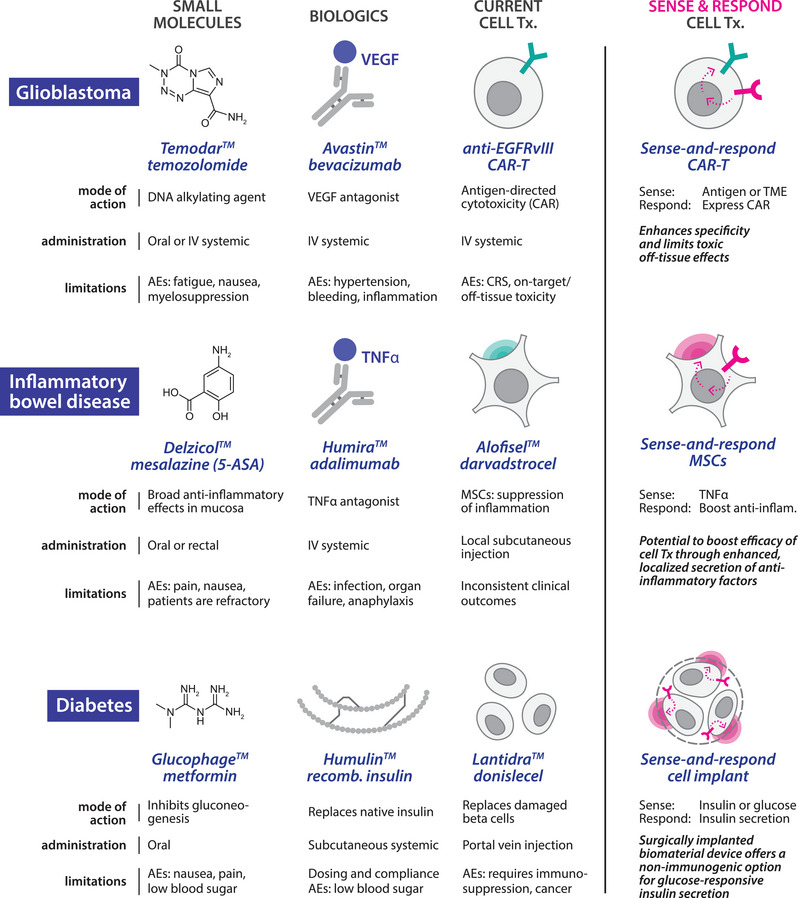
Programming cell therapies to sense and respond is a next phase for therapies. The benefits of small molecule therapies (low cost, simple oral dosing) can be offset by their limiting side effects. Biologics (e.g., monoclonal antibodies or recombinant proteins) have improved specificity over small molecule therapeutics but can suffer from higher costs, poor biodistribution, and invasive delivery. Whilst cell therapies are expensive, they have the potential for more durable responses and reduced need for recurring treatment, but they face significant challenges. In glioblastoma therapy, CAR‐T cell efficacy is impacted by limited antigen choice and on‐target, off‐tissue toxicity. A product using a sense‐and‐respond circuit to tissue‐restrict CAR expression could overcome this challenge. In inflammatory bowel disease, darvadstrocel (unmodified MSCs) is injected into perianal fistulas to reduce inflammation and promote healing but may perform inconsistently in clinical trials. An MSC therapy engineered with a sense‐and‐respond circuit could provide a more robust mechanism of action. In Type II diabetes, careful glucose level monitoring and exogenous administration of recombinant insulin is required. Donislecel is an Food and Drug Administration (FDA) approved therapy that furnishes functional beta cells sourced from a cadaveric donor, but requires immunosuppression that can lead to infections and cancer. Hypoimmune pancreatic islets are in clinical trials and may overcome this challenge. These cells may provide a platform for additionally engineered cells that can further support glucose management through sense‐and‐respond circuits or by reporting on patient health. Icons in pink indicate synthetic sense‐and‐respond function. AEs, adverse events; CAR, chimeric antigen receptor; CRS, cytokine release syndrome; inflam, inflammatory; IV, intravenous; recomb, recombinant; TME, tumour microenvironment; Tx, therapy/therapeutic.

***Advantages of programming therapeutic cells to sense and respond***. The implementation of synthetic sense‐and‐respond circuitry in therapeutic cells represents a paradigm shift in precision medicine, and has the potential to address long‐standing challenges in drug delivery.^1^ Traditional therapeutic modalities, such as small molecules and biologics, can suffer from short in vivo half‐lives and unfortunate side effects. Many cell therapies are challenged by invasive administration requirements for hard‐to‐reach tissues and significant off‐target toxicities, including cytokine release syndrome and on‐target, off‐tissue toxicity.^5^ Synthetic circuits offer a potential solution to these challenges by furnishing cells with the ability to sense disease‐ or tissue‐specific markers and respond by delivering therapeutic payloads with precisely defined spatial, temporal and dose profiles. Beyond enhancing therapeutic precision and minimising side effects, this approach effectively decouples therapeutic mode‐of‐action from the intrinsic properties of the host cell, facilitating programmable, context‐specific responses to be engineered independently from the myriad complexities of native cellular function (Figure 1).

**FIGURE 2:**
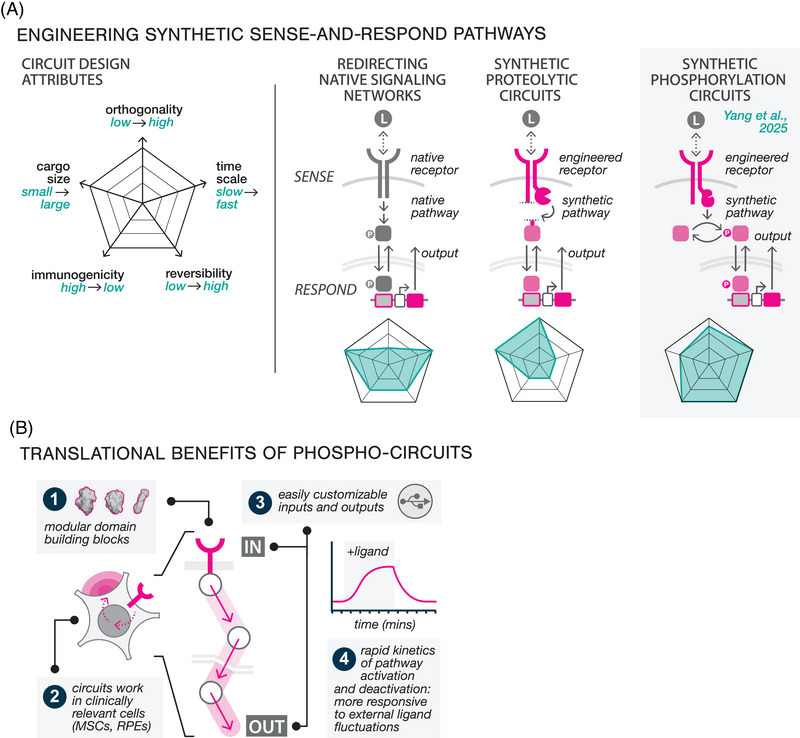
Comparison of sense‐and‐respond technologies. (A) Left: Circuit designs can be compared based on their orthogonality, activation time scale, reversibility, immunogenicity, and cargo size. (Right) A typical sense‐and‐respond circuit converts an extracellular stimulus into a cell‐based response. In systems that redirect native networks, a synthetic response element can convert the endogenous signal into an engineerable output. Synthetic proteolytic and phosphorylation circuits leverage engineered receptors and synthetic pathways to create a complete pathway. Each of these systems has differing circuit design attributes. (B) Phosphorylation‐based circuits provide multiple clinically‐relevant benefits, including modularity, function in clinically relevant cell types, customisable input/output, and a fast response timecale that makes the system more responsive to external stimuli. Icons in pink indicate synthetic sense‐and‐respond function.

***Current state of engineering sense and respond for cell therapies***. Pre‐clinical efforts to engineer synthetic sense‐and‐respond circuits have followed two broad design approaches^9^ (Figure 2A). The first introduces programs that harness the activity of native signalling pathways to drive therapeutic transgene expression.^10^ Whilst these strategies benefit from the speed and robustness of endogenous signalling networks, they are inherently limited by pathway crosstalk, as native signalling components can be readily activated by non‐specific stimuli. A second class of sense‐and‐respond circuits has been constructed primarily from synthetic protein components. Most of these designs rely on receptor‐induced proteolysis to release synthetic transcription factors (TFs) that activate transgene expression or initiate other cellular functions.^7^ These circuits, some of which have recently advanced into clinical trials (NCT06245915), offer several benefits, including the ability to quantitatively tune the response function, and to configure inputs and outputs for diverse indications. However, the inherent non‐reversibility of protease cleavage presents a significant limitation: once the ligand is removed, the cleaved synthetic TF can persist in the cell, resulting in slower rates of activation and deactivation, reducing the circuit's responsiveness to fluctuations in input.

***Phosphorylation‐mediated sense‐and‐respond circuits***. We developed our phosphorylation‐based circuits to retain the tunability and configurability of protease‐based designs whilst addressing their performance limitations^8^ (Figure 2A, right). Although phosphorylation serves as the primary mechanism by which all cells naturally sense fluctuations in their environment, there has been little progress in phosphorylation‐based synthetic circuit design. Our work addressed this gap by developing a streamlined protein domain toolkit for constructing reversible phosphorylation cycles, wherein kinase and phosphatase activities precisely phosphorylate and dephosphorylate a protein substrate. As we demonstrated, these cycles can be linked together into multi‐layered pathways that couple synthetic receptor sensing to downstream cellular outputs such as molecular condensation and transcription. In one demonstration, we engineered a closed‐loop cytokine control circuit that can dynamically suppress activated T cells by detecting tumor necrosis factor (TNF)‐α and secreting interleukin (IL)‐10. Our engineering solution has multiple potential translational benefits (Figure 2B). First, the modularity of our protein domain toolkit allows circuits to function as orthogonal information channels that operate independently from the cell, whilst the use of human‐derived protein domains to construct our circuits minimises the risk of immunogenicity. Second, these circuits function effectively in clinically relevant cell types, facilitating their use across multiple indications. Third, the use of entirely artificial proteins enables seamless reconfiguration of circuit inputs (e.g., disease biomarkers) and outputs (e.g., therapeutic biologics). Finally, rapid and reversible on/off dynamics enabled by phosphorylation offers superior spatiotemporal control over therapeutic response.

***Moving dynamic cell therapies into the clinic***. Whilst our phosphorylation‐based circuits represent a significant advancement in cellular engineering, translating them into clinical applications will require overcoming several key challenges. First, as multi‐protein systems, they necessitate large DNA payloads that exceed the packaging capacity of many commonly used DNA delivery vectors. To enable more efficient delivery, we are developing compact circuit designs compatible with transposon systems and CRISPR knock‐in approaches. A major hurdle for clinical translation is the efficient transfection and transgene expression in primary cells, which can be difficult to manipulate. Currently, we can achieve functional phosphorylation circuit expression in multiple cell types, including retinal pigment epithelium (RPE) cells and mesenchymal stem cells (MSC). However, the manufacturability of cell therapies that incorporate synthetic circuits must be prioritised alongside circuit performance to ensure successful clinical scaling. Through our work, we have identified several factors that critically impact manufacturability, including the toxicity of the DNA delivery method, the size of the genetic payload, and the cellular burden associated with transgene expression, all of which must be carefully considered in order to develop viable, cost‐effective clinical pipelines.

***Conclusions***. The outlook for cell therapies engineered with synthetic sense‐and‐respond circuits is promising, with the potential to address some of medicine's most pressing challenges. Our work establishes a foundation for user‐defined therapeutic responses that behave with natural‐like precision, and we are optimistic that these advancements will drive significant progress in treating a wide range of complex diseases.

## CONFLICT OF INTEREST STATEMENT

A provisional patent application that covers technologies described in this manuscript has been filed by Rice University.
